# Development and Characterization of a Collagen-Based Matrix for Vascularization and Cell Delivery

**DOI:** 10.1089/biores.2015.0007

**Published:** 2015-03-01

**Authors:** Cara E. Ellis, Laura K. Ellis, Ryan S. Korbutt, Erik J. Suuronen, Gregory S. Korbutt

**Affiliations:** ^1^ Department of Surgery, University of Alberta, Edmonton, Canada.; ^2^ Alberta Diabetes Institute, University of Alberta, Edmonton, Canada.; ^3^ Division of Cardiac Surgery, University of Ottawa Heart Institute, Ottawa, Canada.

**Keywords:** angiogenesis, biomaterials, extracellular matrix, tissue engineering

## Abstract

Since the development of the Edmonton protocol, islet transplantation is increasingly encouraging as a treatment for type 1 diabetes. Strategies to ameliorate problems with the intraportal site include macroencapsulating the islets in diverse biomaterials. Characterization of these biomaterials is important to optimally tune the properties to support islets and promote vascularization. In this study, we characterize the cross-linker-dependent properties of collagen-based matrices containing chondroitin-6-sulfate, chitosan, and laminin, cross-linked with 7.5, 30, or 120 mM of 1-ethyl-3-(3-dimethylaminopropyl) carbodiimide and N-hydroxysuccinimide. The swelling ratio was found to be significantly negatively correlated with increasing cross-linker concentrations (*p*<0.0001; R2=0.718). The matrix released insulin in a reproducible logarithmic manner (R2 of 0.99 for all concentrations), demonstrating cross-linker-dependent control of drug release. The matrices with the highest cross-linker concentrations resisted degradation by collagenase for longer than the lowest concentrations (58.13%±2.22% vs. 13.69%±7.67%; *p*<0.05). Scanning electron microscopy images of the matrices revealed that the matrices had uniform topography and porosity, indicating efficient cross-linking and incorporation of the polymer components. Matrices were transplanted subcutaneously in naive BALB/c mice, and the number and size of vessels were quantified using von Willebrand factor staining; matrices with higher cross-linking concentrations had significantly larger capillaries at every time point up to 4 weeks after transplantation compared to the lowest cross-linker concentration group. CD31 staining visualized the capillaries at each time point. Taken together, these data show that this collagen-based matrix is reproducible with cross-linking-dependent properties that can be optimized to support vascularization and islet function.

## Introduction

Islet transplantation is a promising clinical cell-based therapy for treatment of type 1 diabetes.^1–4^ However, despite remarkable progress, the liver implantation site remains far from ideal. Clinical transplantation of islets into the portal vein has been associated with life-threatening intraperitoneal bleeding,^5^ portal vein thrombosis, and hepatic steatosis.^6,7^ The liver may also contribute to the gradual attrition of chronic islet graft function.^8^ Search for a safer alternative site for islet transplantation is therefore desirable and an important issue to address.^9^ Using biomaterials to deliver islets to an alternate site could be advantageous if vascularization was promoted, particularly if an immune barrier could also be incorporated into the device.^9^ Diverse techniques have been attempted to this end, including utilizing a polyethylene terephthalate mesh bag,^10^ a polyurethane foam dressing,^11^ a stainless steel mesh with polytetrafluoroethylene stoppers,^12^ and gelatin microspheres in a collagen-coated polyvinyl bag.^13^ Most devices for islet delivery are based on synthetic polymers, which offer the advantages of complete control over mechanical and chemical properties and lower manufacturing costs^14,15^; however, natural polymers offer significant advantages of their own, including the ability of their degradation by-products to be metabolized.^14,15^ In addition, these natural polymers can be engineered to mimic the properties of the natural extracellular matrix (ECM) to support various cell types, including islets and recruited recipient cells.^16^

Collagen-based biomaterial matrices have been previously used to deliver neonatal porcine islets (NPIs) subcutaneously in a murine model.^17,18^ This matrix contains the copolymers chondroitin-6-sulfate, chitosan, and laminin and is cross-linked with 1-ethyl-3-(3-dimethylaminopropyl) carbodiimide and N-hydroxysuccinimide to support NPI viability and function. The matrix is formed in a planar shape to improve graft oxygenation^10^ and has sufficient mechanical strength to resist the mechanical stress of the subcutaneous site. This is an attractive approach to creating a highly vascularized site for the implantation of islets, particularly because the matrix could be functionalized with growth factors that promote angiogenesis.^10^ Our work has demonstrated that this matrix has no effect on glucose-stimulated insulin secretion and can support NPI viability and function *in vivo.*^17,18^ It is important to characterize any biomaterial to ensure that the material functions in a way that supports the viability and function of the target cells; for a material that is intended for vascularization, these properties would include degradation rate, swelling ratio, and degree of vascularization. Biocompatibility depends not only on the material characteristics but also on the biological system in which the material will be used, and therefore, it is valuable to be able to alter the material properties to suit the applications.^19^ Natural polymers can be tuned by various strategies, including the use of copolymers and controlling the degree of cross-linking of all the polymers.^15^ Additionally, it is known that the topography of a material is important as an alternate signaling mechanism to control many properties related to vascularization, including adhesion, migration, and differentiation.^15,16^ We have previously optimized the copolymers in our collagen-based matrix,^18,20,21^ but the most favorable concentration of the cross-linker remains to be determined. In this study, the cross-linker-dependent properties of the collagen-based matrix are characterized to facilitate the optimization of a biomaterial able to support cellular grafts, such as islets for the treatment of type 1 diabetes.

## Materials and Methods

### NPI preparation

Donor pancreases were obtained from 1- to 3-day-old Duroc cross neonatal piglets from the University of Alberta Swine Research Centre (1.5–2.0 kg body weight), and the islets were isolated and cultured for 5–7 days, as described previously.^18,22^ Briefly, the retrieved pancreases were cut into 1- to 3-mm tissue fragments, then exposed to 2.5 mg/mL collagenase (type XI, C7657; Sigma), filtered through a 500-μm nylon screen, and washed in Hank's Basic Salt Solution (HBSS, H6136; Gibco) supplemented with 0.25% BSA (fraction V, A9543; Sigma-Aldrich), 10 mM HEPES (H4034; Sigma-Aldrich), 100 U/mL penicillin, and 0.1 mg/mL streptomycin (09-757F; Lonza Walkersville, Inc.). NPI were then cultured in nontissue culture-treated Petri dishes containing Ham's F10 tissue culture media (N6635; Sigma-Aldrich) supplemented with 14.3 mM sodium bicarbonate (S233; Fisher), 10 mM D-glucose (DX0145-3; EM Science), 2 mM l-glutamine (G8540; Sigma-Aldrich), 0.25% BSA (fraction V), 50 μM isobutylmethylxanthine (I5879; Sigma-Aldrich), 10 mM nicotinamide (N0636; Sigma-Aldrich), 1.6 mM calcium chloride dihydrate (C7902; Sigma-Aldrich), 100 U/mL penicillin, and 0.1 mg/mL streptomycin (09-757F; Lonza Walkersville, Inc.). The islets were cultured at 37°C for 5–7 days, with the medium changed at the first, third, and fifth days after isolation.

### Preparation of collagen matrices

Collagen matrices cross-linked with 7.5, 30, and 120 mM of 1-ethyl-3-(3-dimethylaminopropyl) carbodiimide (EDC, E6383; Sigma-Aldrich) and N-hydroxysuccinimide (56480; Sigma-Aldrich), containing 0.2 mg/mL chondroitin-6-sulfate (034-14612; Wako Pure Chemical Industries), 1.0 mg/mL chitosan (C3646; Sigma-Aldrich), and 0.1 mg/mL mouse laminin (354232; BD Biosciences, Inc.), were manufactured using previously described methods.^18,22^ High-concentration denatured rat tail type 1 collagen (354249; BD Biosciences, Inc.) was used. Briefly, all components were mixed together in a 50-mL glass tube on ice, with varying concentrations of cross-linker added at the last time. The liquid matrix was then adjusted to a pH of 6.0 for 5 min to initiate cross-linking, then 150 μL of liquid matrix was added to the wells of a 24-well plate; matrices were then cross-linked for 30 min at 37°C before continuing on to the *in vitro* or *in vivo* analyses. Before transplantation, matrices were cultured for 24 h in phosphate-buffered saline (PBS) to ensure excess cross-linker was removed.

### In vitro *measurements of matrix properties*

The masses of the matrices were measured after a 30-min cross-linking period. The matrices were then dehydrated for 1 h in increasing concentrations of ethanol, from 70% to 100%, in periods of 15 min. The masses of the fully dehydrated matrices were measured three times to ensure consistency throughout the test. The swelling ratio (Q) of the matrix was calculated by the following formula:

\begin{align*}Q = \frac { M_c - M_d }  { M_c } \end{align*}

where *M_c_*=cross-linked weight and *M_d_*=dehydrated weight.

To ensure that hormones or growth factors can readily diffuse out of the matrix, 34.7 μg (1 UI) of porcine insulin was added to 1000 μL of liquid matrix in triplicate and then PBS without insulin was added on top of the matrices. All the PBS was removed, and fresh PBS was added at 30 min, 1 h, 2 h, 3 h, 4 h, and 6 h to mimic biologically fast acting insulin. The PBS was subsequently assayed for porcine insulin using a commercial mouse/rat insulin assay (K152BZC; Meso Scale Diagnostics).

### Scanning electron microscopy

Matrices were also taken for scanning electron microscopy (SEM) analysis of the microstructure using the technique described by McEwan et al.^20^ Briefly, to preserve cell morphology, matrices with cells were fixed in 3% glutaraldehyde (Sigma-Aldrich) buffered with 0.1 M PBS for 30 min and then rinsed with PBS three times before ethanol washes. Ten millimeters of diameter matrix samples were dehydrated in 70%, 80%, 90%, 95%, and 100% ethanol solutions for 10 min each. For cross-sectional viewing, samples were fractured after immersion in liquid nitrogen. Samples were sputtered (Hummer VII; Anatech) with a palladium/gold (60:40 palladium:gold) alloy to form a thin coating (3 nm). SEM images were obtained using an accelerating voltage of 1.0 kV to minimize sample damage. Cross-sectional images were obtained using backscattering and secondary electron detectors. Micrographs were evaluated for porosity and pore diameter using ImageJ 1.43u software.

### In vitro *degradation*

The masses of matrices with 7.5, 30, and 120 mM cross-linker concentrations were measured, and then, the matrices were exposed to 25 U of type V collagenase in PBS at 37°C (*n*=3 for each matrix type). At 30 min, 1 h, then hourly up to 10.5 h, the collagenase solution was aspirated and any adsorbed solution removed, and then, the matrices were weighed to determine the *in vitro* degradation rate.

### NPI survival

Apoptosis of the NPIs in the matrices was assessed after 7 days in culture using a commercial TUNEL assay kit (A23210; Invitrogen Molecular Probes). The matrices were fixed in Z-Fixx, paraffin embedded, and then, slides of sections were prepared. After rehydration, antigen retrieval was performed in a sodium citrate buffer (pH 6.0), and then, slides were incubated according to the manufacturer's instructions. The slides were mounted with ProLong Gold Antifade Reagent with DAPI (P36935; Invitrogen Molecular Probes). To quantify the percentage of TUNEL-positive cells, five images of TUNEL and DAPI staining were taken, and then, the images were combined in ImageJ (National Institutes of Health). Separate images of the TUNEL- and DAPI-positive channels were altered to black and white, and then, the number of particles was counted. Each particle was confirmed by two independent reviewers to be TUNEL or DAPI positive visually and the brightness of the color threshold adjusted to only include positive cells. The number of TUNEL-positive cells was subsequently divided by the total DAPI-positive cells to determine the percentage of apoptotic cells in each image.

### In vivo *measurements of matrix properties*

Matrices with varying cross-linker concentrations were transplanted subcutaneously under the dorsal skin of naive BALB/c mice. These matrices were retrieved at 2, 3, and 4 weeks. After retrieval, all collagen-based matrices were fixed in Shandon Zinc Formal-Fixx (6764255; Thermo Fisher Scientific), then embedded in paraffin, and 5-μm sections were prepared. For vascularization, CD31 and von Willebrand factor (vWF) staining was utilized to visualize arterioles and capillaries. After rehydration, antigen retrieval for both CD31 and vWF was performed in the sodium citrate buffer (pH 6.0). All immunohistochemical samples were blocked with 20% normal goat serum for 20 min (0005-000-121; Jackson ImmunoResearch Laboratories, Inc.). Slides were visualized with an Axioscope II microscope equipped with an AxioCam MRC and analyzed with Axiovision 4.6 software (Carl Zeiss). Five images of vWF staining were taken from each of two retrieved matrices for each cross-linker concentration, and the images were combined for particle analysis in ImageJ (National Institutes of Health). Vessels were counted if they were between 50 and 10,000 μm^2^ in size, with a circularity greater than 0.2. Each particle was confirmed to be a capillary-like structure visually and excluded manually if mistakenly included by the software. Images of CD31 staining were also obtained to confirm that the vWF staining identifies the quality and stability of the vessels.

## Results

### Swelling behavior and drug release are cross-linker dependent

**FIG. 1.**
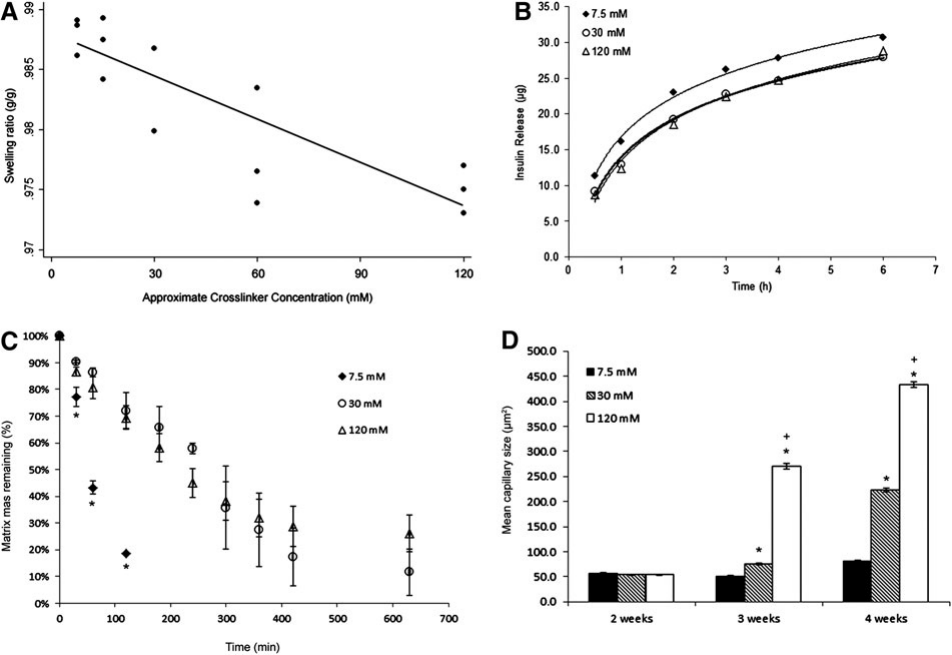
**(A)** Matrices were weighed immediately after the cross-linking period and then dehydrated in increasingly concentrated ethanol solutions. The swelling ratio was calculated as the ratio of the difference between the cross-linked matrix mass and the dehydrated matrix mass to the cross-linked matrix mass. **(B)** One unit of insulin was added to matrices with 7.5, 30, or 120 mM cross-linker concentration, and then, PBS was added on top of the matrices. The insulin diffusion into the PBS was measured at 30 min, then at 1, 2, 3, 4, and 6 h. **(C)** Matrices were exposed to 25 U of type V collagenase. The masses of the matrices were measured at 30 min, then hourly up to 10 h, or until the matrices were completely degraded. **(D)** Matrices were transplanted subcutaneously in naive BALB/c mice and then retrieved at 2, 3, or 4 weeks after transplantation. Matrices were paraffin embedded, then the sections were stained for vWF, and the capillary size was measured using ImageJ. *p<0.05 versus 7.5 mM cross-linking concentration; ^+^*p*<0.05 versus 30 mM cross-linking concentration. All results are shown as mean±standard error of mean. PBS, phosphate-buffered saline; vWF, von Willebrand factor.

The swelling ratio of the collagen-based matrix was significantly correlated with the cross-linking concentration (*p*<0.0001), with a coefficient of determination of 0.718 (Fig. 1A). Increasing the cross-linking concentration resulted in a lower swelling ratio, consistent with hydrogel behavior.

Insulin was diffused from the matrix with all cross-linking concentrations in a reproducible logarithmic manner, with a coefficient of determination of 0.99 for all concentrations (Fig. 1B). By the end of 6 h, a total of 30.62 μg of insulin was released from the matrices with 7.5 mM cross-linker (88% of the total insulin), 27.89 μg of insulin had been released from the matrices with 30 mM cross-linker or ∼80% of the total insulin, and 28.90 μg of insulin had been released from the matrices with 120 mM cross-linker (83% of total). A lower cross-linker concentration seemed to release insulin more quickly, but none of the groups was significantly different.

### *Increased cross-linker concentration gives resistance to* in vitro *degradation*

The matrices with 7.5 mM cross-linking concentration were significantly more degraded as early as 30 min compared to the matrices with 30 and 120 mM cross-linking concentrations (77.20%±3.73% vs. 90.41%±0.57% and 86.76%±1.64%, respectively; *p*<0.001; Fig. 1C). The 7.5 mM cross-linking concentration matrices were also significantly more degraded at 1 h compared to the matrices with 30 mM and 120 mM cross-linkers (43.32%±2.59% vs. 86.32%±1.63% and 80.73%±4.12%, respectively; *p*<0.01) and at 2 h (18.62%±0.23% vs. 72.12%±6.65% and 69.52%±4.56%; *p*<0.01). These low cross-linking concentration matrices were completely degraded after 3 h of exposure to 25 U/mL collagenase. There were no significant differences in degradation between the matrices with 30 and 120 mM cross-linking concentrations at any time point; both groups were too degraded to be measured after 10.5 h.

### Increased cross-linker concentration is associated with increased vascularization

**FIG. 2.**
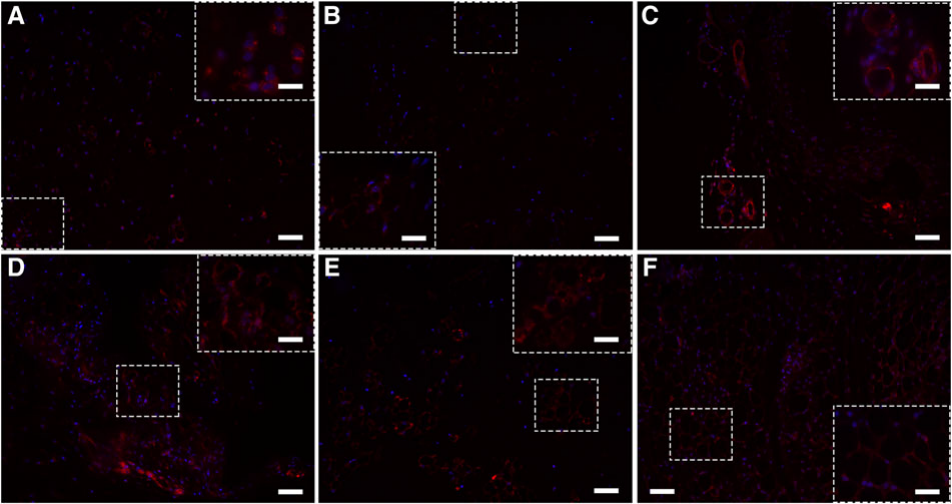
Matrices were transplanted subcutaneously in naive BALB/c mice and then retrieved at 2 or 4 weeks after transplantation. Matrices were paraffin embedded; sections were stained for CD31 (red) and DAPI (blue). Vessels of matrices with 7.5 mM **(A, D)**, 30 mM **(B, E)**, and 120 mM **(C, F)** cross-linker concentrations were visualized at 2 weeks **(A, B, C)** and 4 weeks **(D, E, F)** after transplantation. Scale bars of **(A–F)** are 50 μm; scale bars of high magnification insets are 10 μm.

At 2 weeks after transplantation, there were no significant differences between the capillary sizes in the matrices with 7.5, 30, or 120 mM cross-linker concentrations (*p*=0.054; Fig. 1D). At 3 weeks, the matrix with 120 mM cross-linker concentration had significantly larger capillaries (270.38±4.89 μm) compared to the other two cross-linker concentrations (50.82±1.52 and 75.24±2.32 μm for 7.5 and 30 mM cross-linker concentrations, respectively; *p*<0.05) and compared to the mean size of the capillaries at 2 weeks. Additionally, the matrix with 30 mM cross-linker concentration had a significantly larger mean vessel size compared to the matrices with 7.5 mM cross-linker concentration. Similarly, at 4 weeks, the matrix with 120 mM cross-linker concentration had significantly larger capillaries (433.12±5.12 μm) compared to the other two cross-linker concentrations (81.82±1.20 and 221.68±5.63 μm for 7.5 and 30 mM cross-linker concentrations, respectively; *p*<0.05) and compared to the mean size of the capillaries at 2 weeks. Again, the matrices with 30 mM cross-linker concentration had a significantly larger mean capillary size compared to the 7.5 mM cross-linker concentration matrices (*p*<0.05). All groups had significantly larger capillaries than the earlier time points. These quantitative data are supported by CD31 staining. Two weeks after transplantation, CD31-positive cells and some small vessels can be observed in the matrices with 7.5 mM (Fig. 2A), 30 mM (Fig. 2B), and 120 mM (Fig. 2C) cross-linker concentrations. As the cross-linking concentration increases, larger vessels are visible. Four weeks post-transplantation, all matrices exhibited many CD31-positive vessels, with the matrices with 120 mM cross-linker concentration showing the largest and most plentiful vessels (Fig. 2F) compared to those with 7.5 mM (Fig. 2D) and 30 mM (Fig. 2E) cross-linker concentrations.

### Nanotopography is cross-linker dependent

**FIG. 3.**
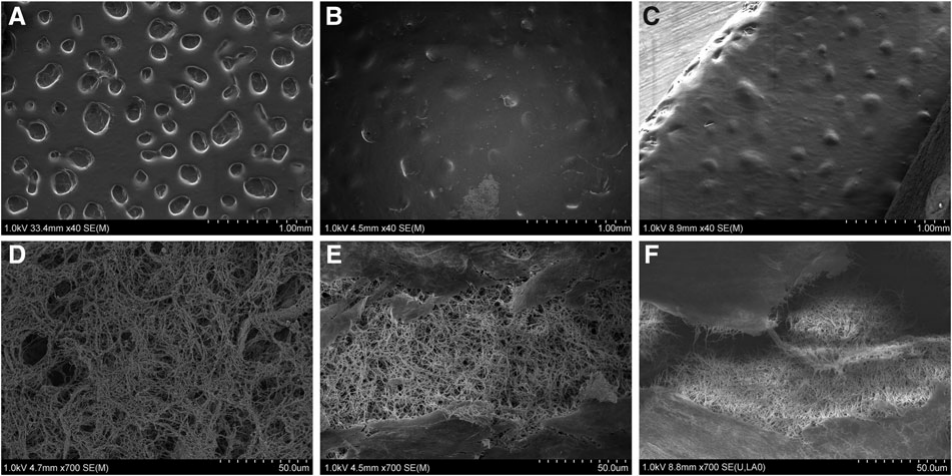
The microstructures of the matrices with 7.5 mM **(A, D)**, 30 mM **(B, E)**, and 120 mM **(C, F)** cross-linker concentrations were examined using scanning electron microscopy. The surface topographies **(A–C)** and interior topographies **(D–F)** were examined.

SEM analysis revealed that the matrix had uniform topography and porosity, indicating efficient cross-linking and incorporation of the different components. At a micrometer level, geometric changes in the surface of the matrices were observed. Matrices with 7.5 mM cross-linking concentration were observed to have micropores with a diameter of 166±6.4 μm (Fig. 3A), whereas matrices with 120 mM cross-linking had microconvexities with a diameter of 148±8.0 μm (Fig. 3C). The nanotopography of the surface of the matrices did not vary with cross-linking concentrations and appeared to be very smooth for a depth of 50±4.3 nm. Below this smooth surface, a randomized network of cross-links was visible. The matrices with 7.5, 30, and 120 mM cross-linking concentrations had significantly different average crosslink densities of 63%±2.2%, 70%±2.5%, and 82%±2.3%, respectively (*p*<0.05 between 120 and 30 mM, and 30 and 7.5 mM; *p*<0.01 between 120 and 7.5 mM; Fig. 3D–F). The average cross-link diameters of all three matrices were not significantly different (137.8±12.0 nm).

### The matrix supports NPI viability at all cross-linker concentrations

**FIG. 4.**
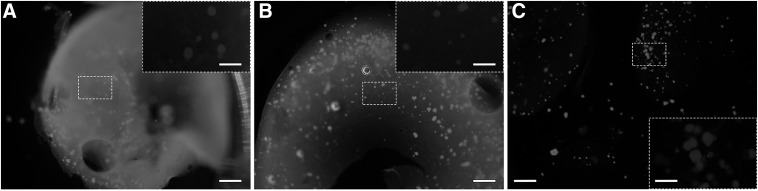
NPIs embedded in matrices with 7.5 mM **(A)**, 30 mM **(B)**, and 120 mM **(C)** cross-linker concentrations were cultured for 7 days, and then, dark field images were taken of the matrices in a six-well plate. Scale bars are 1.6 mm for **(A–C)**; scale bars of high magnification insets are 400 μm. NPIs, neonatal porcine islets.

After 7 days of *in vitro* culture with embedded NPIs, there were visible differences between the matrices with the three different cross-linking concentrations. The group with the 7.5 mM cross-linking concentration was the most opaque and maintained sufficient mechanical integrity for manipulation. There appeared to be pores visible under light microscopy (Fig. 4A, inset). Matrices with 30 mM cross-linking concentration had improved mechanical integrity compared to the 7.5 mM group and were slightly more translucent (Fig. 4B). Interestingly, the addition of NPIs to the matrices with 120 mM cross-linking concentration caused the matrices to lose mechanical integrity after 7 days such that manipulation resulted in the matrix fragmenting into multiple pieces (Fig. 4C). The matrices with 120 mM cross-linking concentration were completely transparent. Intact NPIs were visible in all three matrix groups (Fig. 4, insets).

**FIG. 5.**
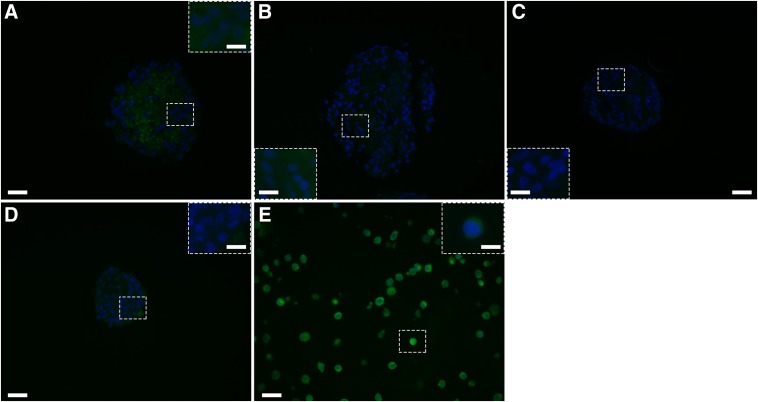
NPIs embedded in matrices with 7.5 mM **(B)**, 30 mM **(C)**, and 120 mM **(D)** cross-linker concentrations and in standard Ham's F10 culture media as a control **(A)** cultured for 7 days, then fixed and paraffin-embedded; sections were stained for TUNEL and compared to TUNEL-positive controls **(E)**. Scale bars of **(A–E)** are 20 μm and scale bars of high magnification insets are 10 μm.

No TUNEL-positive cells were visible in any of the matrices (Fig. 5), indicating excellent support for the NPIs. The control NPIs cultured in the standard Ham's F10 media (Fig. 5A) had the most apoptotic cells as one or two cells per section were TUNEL positive.

## Discussion

These data, combined with our previously described results, demonstrate that this collagen-based matrix has properties reproducibly tunable by the cross-linker concentration and thus could be adapted for various tissue engineering and cell delivery purposes. Our previous work has demonstrated that the matrix with 30 mM cross-linker concentration promotes survival and function of NPIs *in vivo*^18^; data from this study will allow further optimization of the matrix to allow adjustment of the biocompatibility of the system in a variety of situations. All matrices tested have sufficient initial mechanical strength to be easily transplanted and subsequently retrieved if necessary, which may be an important factor when delivering a xenogeneic cell product such as NPIs.^10^

The swelling ratio of the matrix was significantly correlated with cross-linker concentration. Controlling the swelling ratio of a hydrogel could offer control of drug release^23^; this matrix could be functionalized with additional growth factors, such as vascular endothelial growth factor (VEGF), localized immunosuppressive drugs, or anti-inflammatory cytokines or drugs to protect the cell product. This could also be modified by adjusting the concentration of chondroitin-6-sulfate, a glycosaminoglycan (GAG) known to bind and sequester growth factors and cytokines,^16^ if a specific level of cross-linker concentration was required for another aspect of biocompatibility. Insulin was diffused more quickly in matrices with a lower cross-linking concentration, supporting the rationale to adjust the cross-linker concentration as a technique for controlling drug release. In particular, the immunomodulatory and anti-inflammatory cytoprotective factors secreted by mesenchymal stem cells (MSCs) have been shown to protect islets from proinflammatory cytokines^24^; the matrix could have hepatocyte growth factor, fibroblast growth factor, or MSCs themselves added to further protect the islet graft. The properties of the collagen-based matrix could then be tuned to support MSC viability and function.

Matrices with higher cross-linker concentrations better resisted collagenase degradation compared to those with lower cross-linker concentrations. This is related to the swelling ratio as less fluid entering the matrix decreases the exposure to the collagenase solution. All matrices showed sufficient resistance to *in vivo* degradation to allow transplantation,^15^ but it is valuable to have the capacity to control degradation for diverse applications; for example, to match the natural healing or regeneration processes of the recipient tissues.^25^ The number and size of vessels also increase with cross-linker concentration, an advantageous property as these higher cross-linking concentration matrices would have a greater neovascular system to support transplanted cells in the long term. As higher cross-linker concentrations did not have cytotoxic effects on the NPIs, a matrix transplanted with a higher cross-linker concentration, transplanted without a cell population, could benefit from faster more functional vascularization of an ectopic site. However, this remains to be tested *in vivo*.

SEM imaging of the matrix with 7.5, 30, and 120 mM cross-linking concentration revealed uniform microstructure throughout the samples. Interestingly, the micropores visible on the surface of the 7.5 mM matrix do not correlate with increased vascularization, whereas the microconvexities in the 120 mM matrix are related to faster neovascularization *in vivo.* There appears to be a relationship between higher surface energy from cross-linking and the resultant geometric changes and vascularization. The geometric changes lower the surface energy of the matrices; surface energy is an important characteristic of a material that may be more important than topography for guiding cellular adhesion and proliferation.^26^ Although the surface energies of these matrices were not directly measured, the microconvexities of the 120 mM cross-linking concentration matrices are likely a result of higher surface energy that allows for improved cell spreading and ameliorated vascularization.^27,28^ The dense interconnected structure contributes to the mechanical strength of the matrices and may facilitate cell attachment and intracellular signalling.^29^ Additionally, the availability of ECM proteins such as laminin and collagen can provide vital signaling cues and allow ECM receptor interactions; degradation of these proteins can provide substrates as well as space for angiogenesis.^30^ Thus, both the surface energy and topography can potentially be tuned by the cross-linking concentration to support the target cell population, for example, to encourage angiogenesis.

The rapid increase in vascularization between 2 and 3 weeks in the 120 mM matrices is likely due to increasing porosity from biodegradation and infiltrating endothelial cells that remodel the matrix.^31^ SEM imaging of vascularized matrices at varying time points would be helpful in elucidating how the neovasculature forms in the center of the matrix, with or without the presence of proangiogenic factors, such as VEGF. Tuning the porosity to mimic that of the matrices at later time points could also be useful for promotion of angiogenesis. Alternatively, McFadden et al.^32^ demonstrated a technique for *in vitro* prevascularization of a collagen-GAG matrix using human umbilical vein endothelial cells and MSCs. Additionally, a matrix could be transplanted subcutaneously for longer than 4 weeks without a cell population to establish vascularization in the desired site before the delivery of the graft. As this matrix cross-links at 37°C, a liquid scaffold could be noninvasively injected to fill the subcutaneous space.^17,33^ The cytotoxicity of higher cross-linker concentrations in this context requires further exploration. These could be promising approaches for engineering a matrix that can support NPI in a subcutaneous space that lacks sufficient initial vascularization to support the graft.^10^

The natural ECM of the pancreas is highly complex and varies between species.^16,34^ Although the specific roles of the various proteins on endocrine function are not well understood, it is well known that isolated islets suffer from anoikis, a form of apoptosis.^35^ The lack of TUNEL-positive islet cells within the matrix supports the rationale for including collagen, laminin, GAG, and polysaccharide such as chitosan, to prevent this form of apoptosis by presenting a scaffold with similar components as the natural pancreatic ECM.^16,35^ Continued research on the specific interactions between NPIs and these matrix components is warranted to further ameliorate NPI survival and function.

## Conclusions

Overall, these data indicate that this collagen-based matrix with the incorporation of chitosan, chondroitin-6-sulfate, and laminin is reproducible, tunable, and may be used to create a vascularized site for cell delivery, specifically for but not limited to NPIs. This matrix offers the potential for creating a vascularized ectopic site that can be thoughtfully designed for diverse applications.

## Abbreviations Used

ECM: extracellular matrix
GAG: glycosaminoglycan
MSCs: mesenchymal stem cells
NPIs: neonatal porcine islets
PBS: phosphate-buffered saline
SEM: scanning electron microscopy
VEGF: vascular endothelial growth factor
vWF: von Willebrand Factor
